# Morphological and functional trait divergence in endemic fish populations along the small-scale karstic stream

**DOI:** 10.1186/s40850-023-00191-8

**Published:** 2023-12-11

**Authors:** Elif Acar, Nehir Kaymak

**Affiliations:** Faculty of Science, Department of Biology, Akdeniz University, Antalya, Türkiye

**Keywords:** *Pseudophoxinus antalyae*, Geometric and linear morphometrics, Functional traits, Phenotypic plasticity, Morphological variation, Düden Stream, Türkiye

## Abstract

**Background:**

Organisms with broad distribution ranges, such as fish, often exhibit local ecological specializations based on their utilization of food and habitat. Populations of species that live in different habitat types (lotic vs. lentic) show morphological variations. However, the phenotypic differences of endemic fish populations in a small karst river basin under anthropogenic pressure are still not fully understood. In this study, the functional traits and morphological variations of the populations of endemic *Pseudophoxinus antalyae* Bogutskaya, 1992, in the Düden Stream basin, which is subjected to various anthropogenic disturbances and habitat types in southwestern Anatolia of Türkiye, were examined using linear measurements and geometric morphometric analysis.

**Results:**

Differences have been identified in functional traits, particularly those related to food acquisition between populations. Results of both univariate and multivariate analyses revealed significant differences in body shape and size among populations living at sites along the stream with different habitat and environmental characteristics.

**Conclusions:**

The reason for these differences determined in the morphology and traits of the populations may depend on habitat types, ecological, or environmental, and obstruction of gene flow. More detailed studies are needed to explain the mechanisms (genetic and ecological) that cause these differences.

## Background

The variability of intra-specific functional traits is highly important for understanding ecological and evolutionary dynamics [1]. Functional traits emerge in an organism through its performance (growth rate, survival, and reproduction) and the influence of ecological processes [2]. An organism’s phenotype is determined by the interaction of functionally integrated traits and undergoes changes through genetic-epigenetic responses to constantly changing environmental pressures [3]. When environmental conditions differ, individuals of the same species often differ in phenotype, thus increasing their fitness in the local environment [4]. These phenotypic differences can occur through phenotypic plasticity and/or local adaptation. The phenotypic plasticity is defined as the ability of organisms to produce distinct phenotypes in response to environmental variation without genetic change [5]. Local adaptation is the process by which phenotypic variation is generated by genetic differences and may be hindered by gene flow [6]. Many freshwater fish species utilize a rich diversity of habitats and exhibit high differences in body morphology and functional traits due to community interactions (such as food type, predators, and competition) within habitat characteristics (current velocity, water depth, water chemistry, substrate type) [3, 7].

Within a river system, individuals of a species population can freely move along the river network [8], allowing for ongoing gene flow among populations. The homogenizing effect of gene flow can impede local adaptation caused by natural selection and constrain phenotypic differentiation [9]. However, in some cases, despite gene flow, phenotypic plasticity can lead to differentiation [10, 11]. River connectivity can be disrupted due to natural (waterfalls, etc.) or anthropogenic factors (dams, hydroelectric power plants), creating biogeographic barriers among fish populations [12]. Species populations can face isolation through natural or anthropogenic barriers, which can lead to reduced gene flow between areas above and below the barrier [13, 14]. This isolation can lead to genetically distinct populations due to the resulting population bottlenecks and inbreeding [15]. It has been reported that long-term isolation processes due to natural or artificial barriers can also lead to morphological differences among populations [11, 16, 17]. However, some studies reported that fish could rapidly respond to ecological changes resulting from anthropogenic disturbance [11, 18, 19].

**Fig. 1:**
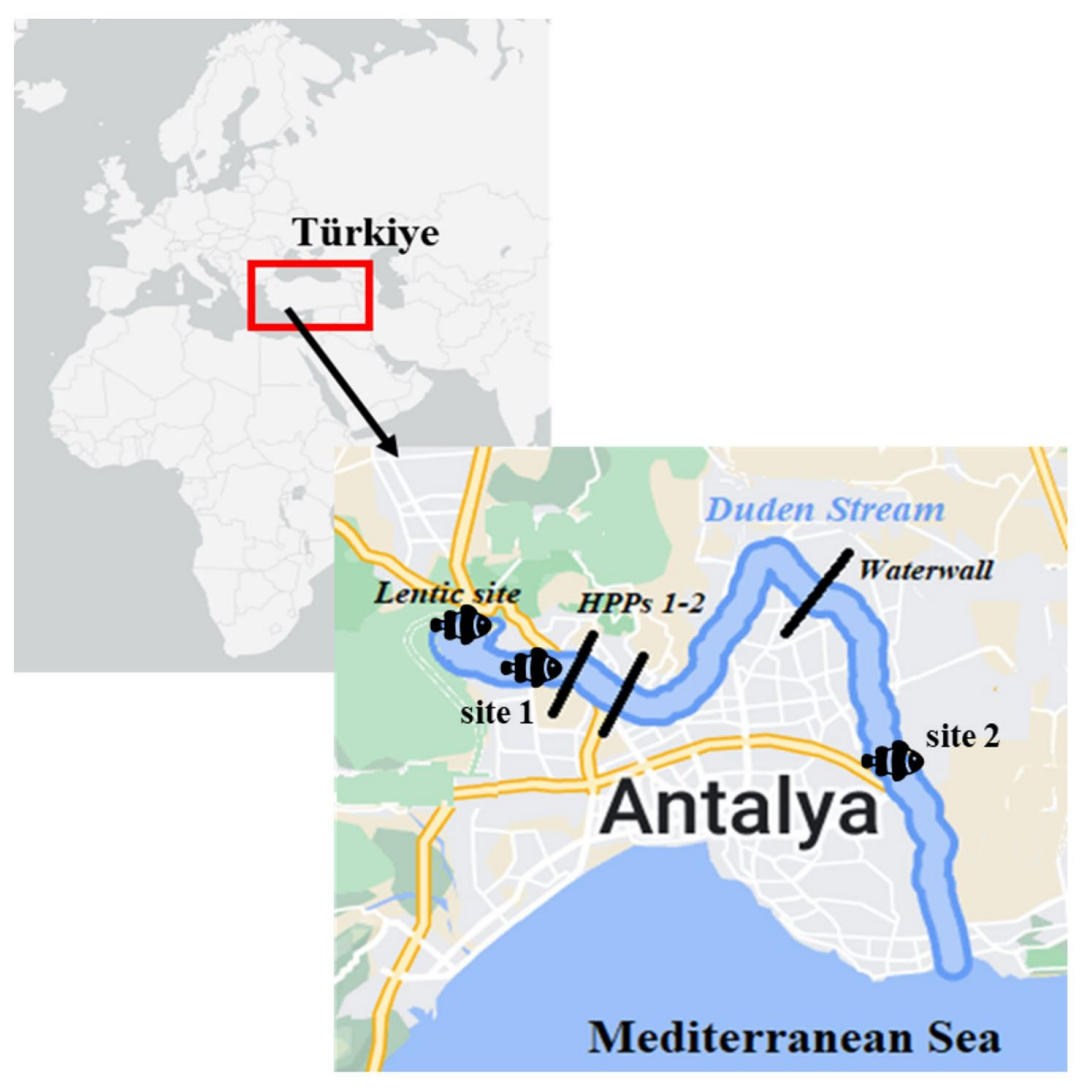
Satellite image of the Düden Stream and the position of the lentic site and two lotic sites (site 1 and site 2) (HPPs 1–2 = two Hydroelectric Power Plants)

Düden Stream is a small karstic freshwater basin located in the southwestern Anatolian of Türkiye. Due to the presence of numerous sinkholes, this small karstic basin possesses an interesting hydrogeomorphology. This small freshwater basin and its biological diversity are significantly threatened due to long-standing anthropogenic activities such as pollutants from agricultural practices, domestic and industrial wastewater discharges, canalization process, and the presence of two hydroelectric power plants [20, 21]. Besides, an invasive species (*Carassius gibelio*) has been also introduced in this stream (N. Kaymak personal observation). *Pseudophoxinus antalyae* (Cypriniformes: Leuciscidae), is an endemic species to the Düden Stream basin [22]. The metapopulation of this small-bodied fish species is under threat due to intense anthropogenic pressures, and it is listed as “Vulnerable” on the IUCN Red List of Threatened Species [23]. The subpopulations of this species have become isolated from each other along Düden Stream due to the presence of two hydroelectric power plants (HPPs, constructed in 1966, and 1987, [24]) and a waterfall (the upper Düden) (Fig. 1). Considering all these anthropogenic, ecological, and hydrogeomorphological conditions in the basin, it is crucial to understand how populations of an endemic species with such a narrow distribution respond to natural and anthropogenic environmental changes and to assess the consequences of habitat degradation on the species evolution. Here, we investigated functional traits and morphological variations in subpopulations of endemic *P. antalyae* in the anthropogenically disturbed Düden Stream basin. Additionally, the effects of site and sex on these variations were also tested. This study allowed predicting how functional morphology patterns of endemic fish populations respond to spatial differences along a stream.

## Methods

### Study area and sampling

Düden Stream is 14 km long and originates from the karst Kırkgöz Springs and falls into the Mediterranean Sea (Fig. 1). While most of the water coming from the spring in the upper basin of the river was channeled for the Kepez Hydroelectric Power Plant (HPPs), some of it goes underground through sinkholes and karst waterways in permeable travertines [24] The canalized water was connected to the natural stream channel before the waterfall (the upper Düden waterfall) in the lower basin of the river. The small amount of water that remains outside the channel many times disappears into the mouth of a cave and passes underground for several kilometers before emerging again in front of the waterfall. The water coming from both the canal and the underground merges before the waterfall (the upper Düden waterfall) and flows to the shore as a stream without sinking again. It then flows into the Mediterranean as a waterfall (Lower Düden Waterfall) over a 40-meter-high cliff. The Düden Stream mostly flows through Antalya city center. Annual precipitation is 856 mm, and the mean annual flow of the stream is 23.8 m3/s [20].

Fishes were captured from three different segments of the Düden Stream basin: the littoral zone of the Kırkgöz Spring (hereinafter referred to as the lentic site), one site located in the Düden Stream and above the HPPs (site 1), and the other site located below the two HPPs and waterfall (site 2) (Table 1; Fig. 1). The sites 1 and 2 were in the stream channel, hence they represent the lotic ecosystem (Table 1). These three sampling sites also were different with respect to geomorphological features, water quality parameters [20, 21], vegetation composition, and fish fauna (N. Kaymak personal observation). The lentic site (Table 1) was located at an altitude of 250–300 m from the sea and covers an area of 45.000 m^2^. This site consists of swampy areas and is densely covered with riparian, emergent, and submerged plant species. According to the heavy metal pollution index (HPI) results [21], it was determined that the water quality of the lentic site was “good” (43.99). Lotic site 1 was the man-made stream channel which was in the upper basin of the Düden Stream, and classified as “poor” (72.53) according to the HPI [21].

**Table 1:** Study site characteristics (habitat type and coordinates) of the Kırkgöz - Düden Stream basin (N = the number of fish individuals caught per site, HPI = heavy metal pollution index)

Sites	Habitat type	Coordinate	Width	HPI	Substrate type	Vegetation	Stream Velocity (m/s)
Kırkgöz Spring	Lentic	37˚06’33.79’’N 30˚34’54.12’’E	45.000m^2^	Good	Sand, Gravel, and Pebble	Presence	-
Site 1	Lotic	36˚58’05.06’’N 30˚37’18.15’’E	5.6 m	Poor	Concrete	Absence	1.84
Site 2	Lotic	36˚54’19.76’’N 30˚45’54.25’’E	49 m	Very Poor	Sand and Mud	Presence	3.59

Lotic site 2 (Table 1) was in the main channel of the lower basin of the stream and Antalya city center which was under the influence of urbanization, agriculture, and industrial activities. Therefore, the HPI was also quite high, and the water quality was classified as “very poor” (242.13) [21]. Riparian vegetation (mostly herbaceous plants) was well developed, and submerged, and emerged plants were scattered in patches in the water.

We sampled fish (Table 2) from three different sites between at the end of May to September 2022 using fyke-nets with a 12–35 mm mesh size. Collected fish were anesthetized with tricaine methane sulfonate (MS-222), and then fixed in 10% formalin and transferred to 70% ethanol for storage. The total 120 individuals were sampled from each site (Table 2). Linear and geometric morphometric methods were used to estimate the variation of morphology and the functional traits between *P. antalye* subpopulations. Photographs of the lateral left side of each fish individual were taken against a centimeter scale using a digital camera (Nikon® D90) attached to a tripod at approximately 50 cm above the sample. Sex identification was performed from these photographs. Because the fins and caudal peduncle of some species of this genus are known to be markedly sexually dimorphic (the male has longer pelvic and pectoral fins and a thinner caudal peduncle) [25, 26].

**Table 2:** Fish total length (cm) and sample size per locality and date (Ns = number of specimens per date, Nt = total number of specimens per site)

Sites	Sampling Dates	Ns	Nt	TL (cm)	
				Mean	Range
Kırkgöz Spring	28.05.2022	5	51	9.54	6.4–16.2
	18.06.2022	11			
	23.07.2022	14			
	27.08.2022	21			
Site 1	28.05.2022	0	25	8.63	6.8–10.3
	18.06.2022	0			
	23.07.2022	10			
	27.08.2022	15			
Site 2	28.05.2022	0	44	7.62	6.1–16.3
	18.06.2022	9			
	23.07.2022	13			
	27.08.2022	22			

The distance between all specimens and the camera was maintained to ensure that the camera position was consistent between specimens, and all individuals were dried before photographing. Photographs were converted into .tps files, using tpsUtil software [27]. Both geometric and linear morphometric measurements were recorded for each sample by the same person using the tpsDIG2 software [27]. In order to prevent measurement results from differing due to the “measurer effect”, it is often recommended that all specimens be measured by the same person and with the same hand, especially in population-level studies [28].

### Functional traits measurements

A total of 10 linear morphometric measurements were used to calculate 8 functional traits of *P. antalyae* populations. Linear measurements were standard length (Sl), body width (Bw), body depth (Bd), head length (Hl), head depth (Hd), eye diameter (Ed), snout length (Snl), mouth depth (Md), mouth width (Mw), and gut length (Gl) (for details, [29]). Selected ecomorphological measurements are associated with different functional groups such as the feeding habits, trophic status, swimming ability and habitat preference of fish. Since these functions are complex processes, they cannot be described using a single measurement or trait [30]. Additionally, the morphometric measurements and traits used here are the most important and widely used variables in distinguishing between populations of species [31]. A digital calliper was used only for mouth depth, mouth width, and gut length measurements. To minimize any variation resulting from allometric growth, data was standardized according to the following formula [32]:

Madj = M(Ls / Lo)b.

where M: actual measurement, Madj: size adjusted measurement, Lo: standard length of fish, Ls: overall mean of standard length for all fish from all samples in each analysis. Parameter b was estimated for each character from the observed data as the slope of the regression of log M on log Lo, using all samples. This transformation best reflects shape variation among groups independently of size factors. Measurements were then converted into eight complementary functional morphological traits that were closely related to food acquisition and locomotion: compression index (Cl), relative gut length (rGl), eye size (Es), gape size (Gs), relative head length (rHl) and depth (rHd), relative snout length (rSnl) (Table 3). We derived these functional traits and its formulas from previous studies [2, 33, 34] (Table 3).

**Table 3:** The eight functional morphology traits and formulas

Functional traits	Code	Measure
Compression index	Cl	Bd² ∕(Sl×Bw)
Oral gape surface	OGS	Mw×Md×Bw×Bd
Relative Gut length	rGl	Gl/Sl
Eye size	Es	Ed/Hd
Gape size	Gs	(MdxMw)/Sl²
Relative head length	rHl	Hl/Sl
Relative head depth	rHd	Hd/Sl
Relative snout length	rSnl	Snl/Sl

### Geometric morphometric analysis

**Fig. 2:**
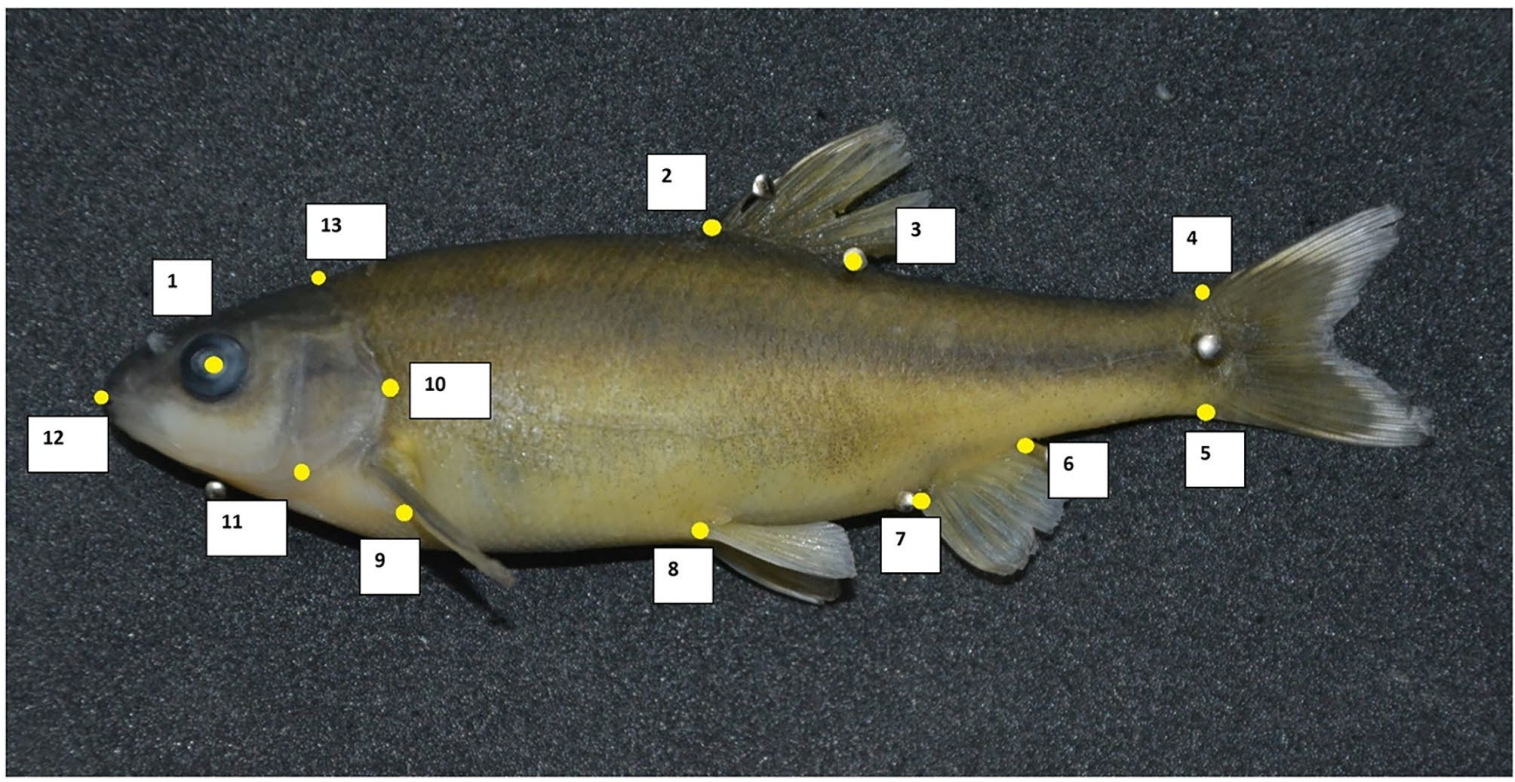
Anatomical landmark digitized in yellow on the left side of *P. antalyae* female: 1: Centre of the eye, 2: anterior point of dorsal fin base, 3: posterior point of dorsal fin base, 4: dorsal point of peduncle-caudal fin junction, 5: ventral point of peduncle-caudal fin junction, 6: posterior point of anal fin base, 7: anterior point of anal fin base, 8: anterior point of pelvic fin base, 9: posterior point of pectoral fin base, 10: the pointed-posterior tip of the operculum, 11: the antero-ventral tip of the suboperculum, 12: mouth tip, 13: dorsal head-body junction

Thirteen landmarks along the entire fish were identified based on a previous study [35] (Fig. 2), and digitized x and y coordinates of these landmarks were generated using the TpsDig 2 program. Raw landmark coordinates were subject to a Procrustes superimposition using General Procrustes Analysis (GPA, least squares method) to remove effects of size, position, and orientation from the raw coordinates and standardize each specimen to unit centroid size [36].

The only variation after this process is particularly shaped variation [37], and this also allows shape comparisons free from allometric growth associated with early ontogeny between populations [38]. The centroid size is used as a measure of whole-body size, defined as the square root of the total square distances between each landmark and the configuration center from the GPA [39, 40]. In this study, centroid size was used as our measure of body size, as it correlated with the standard length of fish samples (Pearson’s, r = 0.78; *P* < 0.001). All these processes were performed using MorphoJ version 1.05f [41].

### Data Analysis

According to the Mann-Whitney U Test results (*p* > 0.05), sexual dimorphism was not determined in 10 linear morphometric measurements, and therefore, functional trait analyses were performed on both sexes combined. To determine how functional traits of the populations varied spatially 8 traits between *P. antalyae* populations were compared using Kruskal-Wallis followed by pairwise Dunn’s post-hoc tests because functional trait values were not normally distributed according to the Shapiro-Wilk test.

Normality and homogeneity assumptions of the variances of centroid size values were evaluated with Shapiro-Wilk and Levene tests, respectively (*p* > 0.05). The differences between sites and sexes for centroid size were analyzed through a two-way Analysis of Variance (ANOVA). Following this analysis, comparisons were made using a Tukey post-hoc test of differences. Principal Component Analysis (PCA) was first used to analyze the shape variation within the entire sample. PCA reveals both the amount of variation and the shape variation associated with each component using Procrustes coordinates created by optimally overlaying each sample on the mean fish shape [42]. The multivariate regression of the Procrustes coordinates as shape variables (with PC axes that most explain the total variation) on the log-centroid size values as a body size variable was performed to analyze the ontogenetic allometry. A multivariate analysis of covariance (MANCOVA, with 9999 permutations) was performed to test whether significant changes in body shape are associated with sites and sexes. For MANCOVA, PC scores (those that explain at least 1% of shape change) served as dependent variables, sites and sexes served as the fixed effect (independent variable), and log-centroid size as covariate. MANCOVA was followed by Wilks’ λ test to determine the degree of shape difference explained by the independent variable.

Discriminant analysis (DFA) was used to further quantify and visualize the inter-population differences in body shape. DFA, which maximizes intergroup variation compared to within-group variation, is used to identify the most extreme examples and the most important discrimination features under the control of predefined important factors [43]. DFA analysis was re-run to focus only on the morphological differences between populations in the lotic sites. In addition, the “Jackknife Groupings” test was used for group assignments [36, 44]. Procrustes data was projected into a thin plate spline (TPS), which visualizes shape changes as a heat map as one sample deforms (changes) into another (i.e. deformation of landmarks relative to mean shape; [44]).

## Results

### Variation in functional traits

**Fig. 3:**
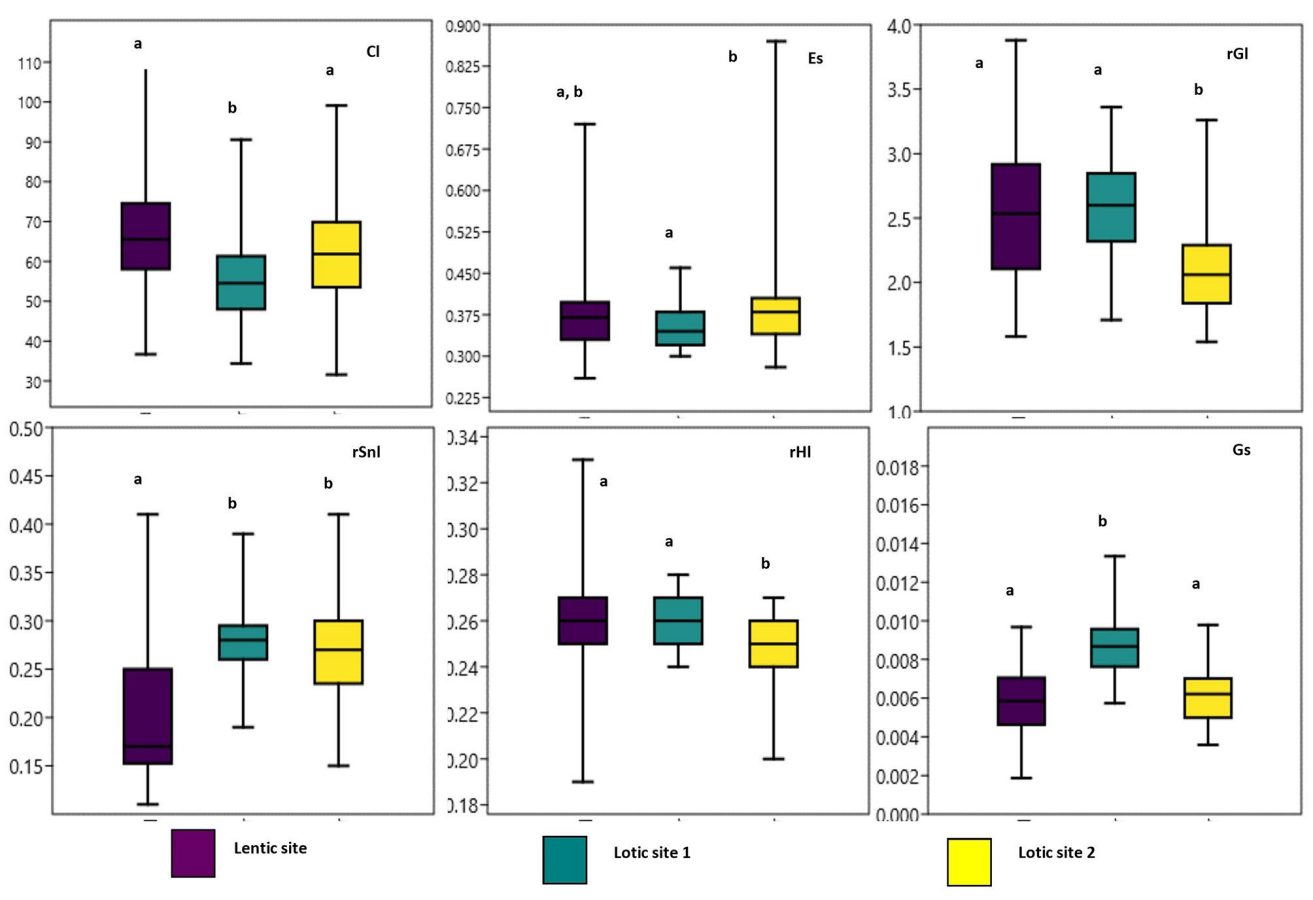
Box plot comparing the functional traits of three populations of *P. antalyae*. Different letters represent statistical differences for each comparison

Functional traits showed significant differences between *P. antalyae* sub-populations. Population from lotic sites had higher rSl than that from lentic site, while Es of the population lotic site 1 was lower than that of the population lotic site 2 (Fig. 3). Besides, the population from lotic site 1 was statistically different from the other two populations in terms of Cl, and Gs. Although rGl, and rHl were considerably smaller in the population from lotic site 2, there was no statistical difference among populations for the rHd (Fig. 3).

### Variations in body shape and size

While there was no significant difference in the centroid sizes of populations from the lentic and lotic site 1, ANOVA revealed that the fish caught from the lentic site had the largest, and the fish from lotic site 2 had the smallest size (F_2,116_ = 10.3, *P* < 0.001). The centroid size was not statistically different among the sexes (F_1,116_ = 0.138, *P* = 0.711), and the interaction between sites and sex was not significant (F_2,116_ = 0.131, *p* = 0.878).

The results of the MANCOVA (Table 4) revealed a significant body shape variation among sites, while the differences in shape were not due to sex. Additionally, the combined effect of both variables (sex*site) on the shape of the body was found to be insignificant. The “Jackknife Groupings” test revealed that populations (at the lentic site, lotic sites 1 and 2) were grouped into the classification accuracy of 84.2%, 88%, and 88.6%, respectively.

**Table 4:** Results of MANCOVA for the analysis of shape variation and its association with centroid size (i.e. shape allometry) in *P. antalyae*

Source of Variation	df_1, 2_	Wilks’ Lambda	F	*p*
Sex	6, 107	0.89	2.19	0.049
Sites	12, 214	0.403	10.27	< 0.001
Sites*Sex	12, 214	0.883	1.15	0.324
Centroid size	6, 107	0.754	5.83	< 0.001

**Fig. 4:**
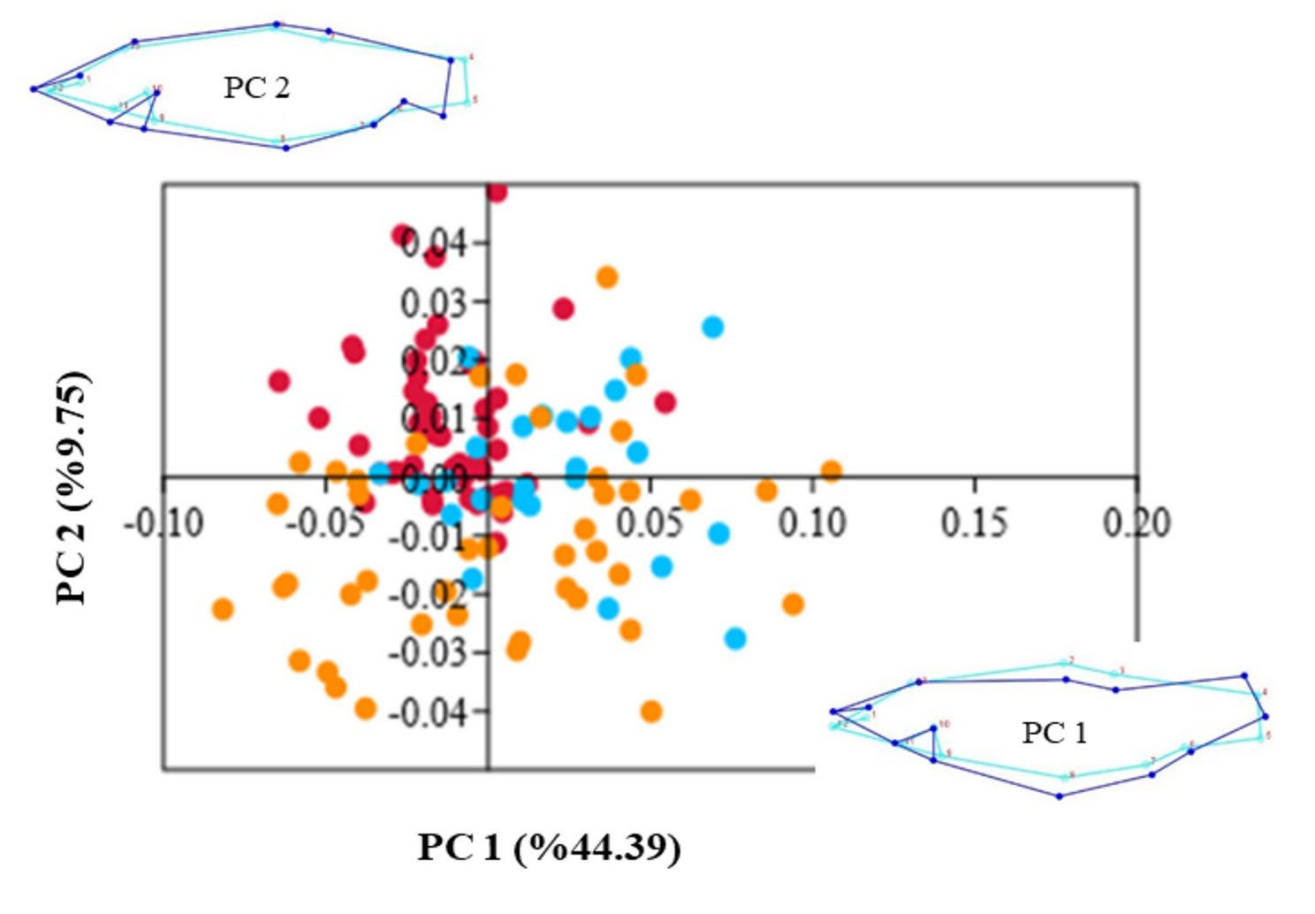
PCA and wireframe of *P. antalyae* from three sites (red dots represent “lentic site”, blue “lotic site 1”, orange “lotic site 2”)

The analysis of PCA revealed that PC1 (44.39%) and PC2 (9.75%) together accounted for 54.14% of the total variation of body shape in *P. antalyae* (Fig. 4). The PC1 axis is strongly negatively correlated with landmarks of the central portion of the body (2, 3, and 8), however strongly positively correlated with landmarks of the caudal peduncle (4, and 5) and mouth (12). In addition, the PC2 axis has negative and positive correlations with landmarks 5 and 7, respectively. Individuals from the lentic site were distributed mainly on the negative side of the PC1 and the positive side of the PC2. While there was no significant shape-size relationship throughout PC1 (r² = 0.007; *p* = 0.359), a slight shape-size relationship was found for PC2 only in the lentic population (r² = 0.31; *p* < 0.001) for ontogenic allometry.

**Fig. 5:**
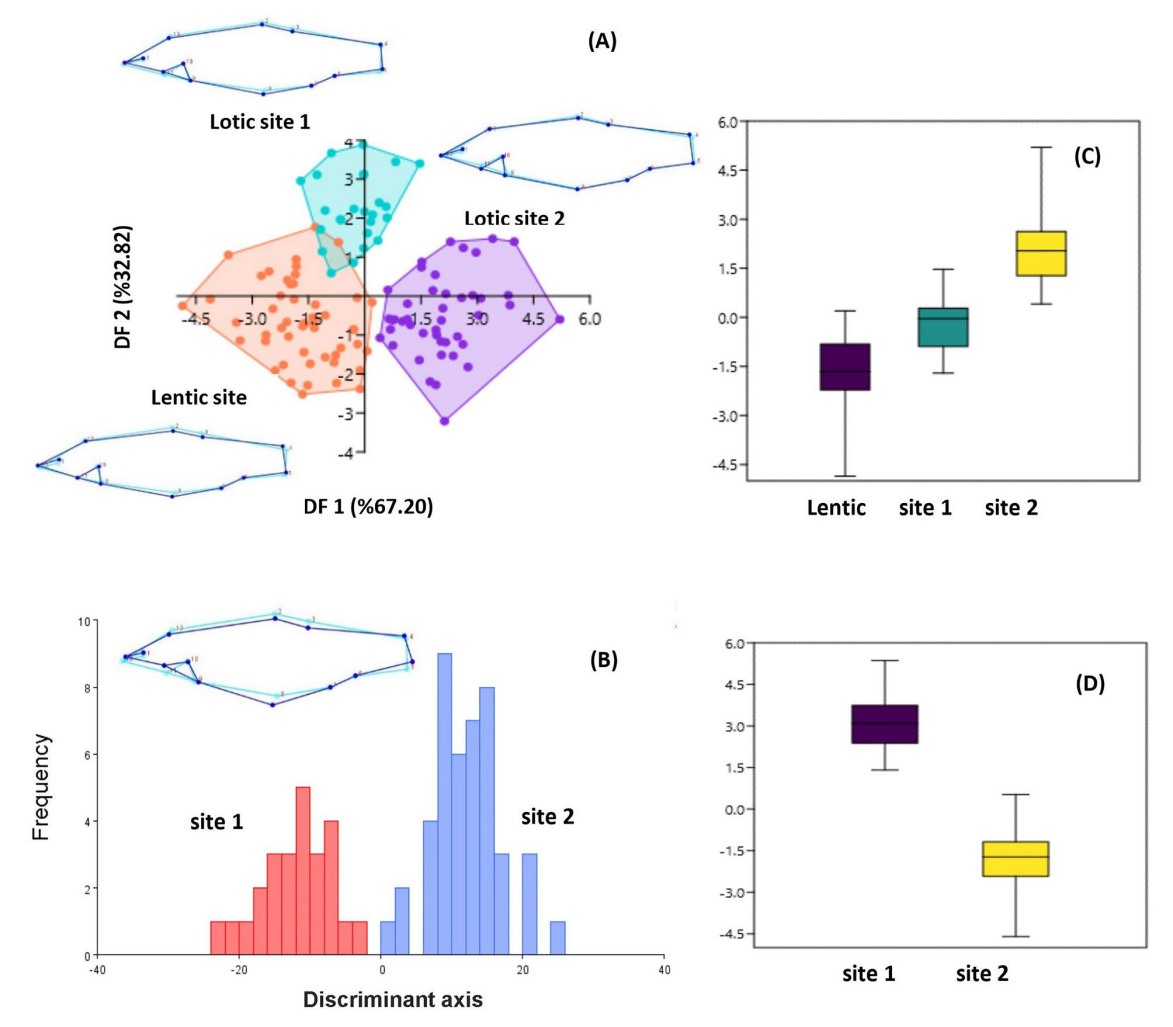
DFA analysis and wireframe showing the shape variation of *P. antalyae* populations depending on all sites (A) and lotic sites (B). C and D are box plots showing the ANOVA results for DF1 (sites 1 and 2 represent the lotic system)

DFA analysis indicated that populations from the three sites differed from each other in shape, although there were partial similarities among the populations from lentic and lotic site 1 (Fig. 5A and B). The DF1 axis, which explains 67.20% of the change in body shape, correlated negatively with landmarks 1, 8, and 13, and positively with 2, 11, and 12 which are mostly associated with the head part of the body. The DFA bi-plot revealed that the shape of the population located lotic site 2 was distinct from the other two populations based on these landmarks. When the DFA analysis re-run, the body shapes of the two populations from lotic sites were clearly different from each other, explained by DF1 with 100% variation. The DF 1 axis was tested against two independent variables: all sites (ANOVA, F = 7.961, *P* = < 0.0001) and lotic sites (ANOVA, F = 18.295, *P* = < 0.0001) were responsible for shape variation among populations (Fig. 5C and D).

**Fig. 6:**
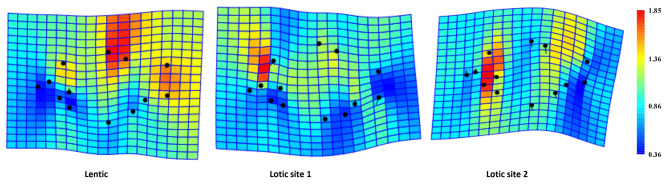
Shape variation of *P. antalyae* populations depicted by thin-plate spline using a deformation heat map

Morphological differences between populations obtained by DFA were consistent with the shape analysis of the TPS heat map (Fig. 6). This high deformation was presented by dark red spots which represent the population shape protruding beyond its mean shape on the heat map of the landmarks. The blue “cold” spots in the TPS grid represent where the population shape shrinks relative to the average shape. The deformation pattern became more complicated in populations from lentic and lotic site 1 relative to those of lotic site 2. The population from the lentic site had a narrowing in the head region, but an increase in body depth and caudal peduncle, while the population from lotic site 1 had a narrowing in the body depth and caudal peduncle, but also an increase in the head-body connection. The population from lotic site 2, on the other hand, was characterized by a broad head, and a slightly narrowed body and caudal peduncle (Fig. 6).

## Discussion

### Functional trait variation

The effect of the environment on organism phenotype will affect its function and functional traits of the organism in the ecosystem [34]. Some traits such as relative gut length (rGl), eye size (Es), and gape size (Gs) are all related to food acquisition [45]. Particularly, gut length is related to the type of food source consumed by the fish. If fish fed less digestible food types developed relatively longer guts compared to those fish fed an easily digestible diet [46]. Individuals from lotic site 1 had a long gut length, larger gape size, and small eyes, which may facilitate access to a high amount of larger and less digestible food sources (mostly herbivorous) at one time [34]. In this case, large eyes are not needed. On the contrary, individuals lotic site 2 have a short gut, small mouth, and large eyes assured that they might have better food detection ability [47], and can secure more easily digestible food resources (mostly carnivore) successfully in an ecosystem with fast-flowing and turbid water, intense interspecific competition [48] Because two cyprinid fish species (*C. gibelio* and *Cyprinus carpio*) were caught with this endemic fish together at the lotic site 2. The snout length of individuals in lotic sites is longer than that of the lentic system, which means that fish in fast-flowing lotic sites have more ability to detect and catch prey. This means that fish in the lotic systems can have a variety of trophic and sensory abilities [33, 49]. As a result, these spatial variations in functional traits of *P. antalyae* were mostly related to food intake and may have switched their diet to the most abundant resource to maximize energy intake. This species is omnivorous and consumes both insect larvae and aquatic plants (N. Kaymak personal observation from gut content). This characteristic of *P. antalyae* may cause its survival and successful adaptation in modified and anthropogenically disturbed local habitats along the Düden Stream.

### Body size and shape variation

Geometric morphometry analyses provided an opportunity to investigate the phenotypic diversity of *P. antalyae* populations along the Düden Stream in response to different ecological conditions. Although the univariate analysis revealed that centroid size differed between populations, this difference was not related to ontogeny (according to regression analysis). Since this variation cannot be explained by fish length, size differences may be related to ecologically driven selection [50]. *P. antalyae* subpopulations exhibited spatial functional and morphological variation. Intraspecies differences in phenotype were likely a product of exposure to varying selective pressures imposed by ecological heterogeneity within the system [37]. The factors that may cause inter-population morphological shape variation are discussed in detail below.

Although the DFA and PCA bi-plots showed partly overlapped between the body shapes of populations from the lentic and lotic site 1, the MANCOVA and deformation heat map results confirmed that the body shape of the two populations was different from each other. Both sites represented different habitat types: lentic and lotic. The steady-unsteady swimming model predicts that lotic populations will show a more aerodynamic form than lentic population [51]. Fish from lentic habitats are typically characterized by a deeper body, a larger caudal area, and a smaller head [52, 53]. This general body structure facilitates sudden acceleration and increases maneuverability [54]. The findings from this study were consistent with previous studies because individuals from lentic site had a deeper body (higher body height), smaller head, and longer dorsal and pelvic fin bases, whereas individuals from above the barrier generally had a narrower body and caudal peduncle. The finding is further supported by low compression index (Cl) values observed in individuals from above the barrier, as a low value represents a dorsa-ventrally compressed body shape [34]. Similar morphological patterns were also common in different cyprinid fish species [55] and populations of small-bodied carp, *Cyprinella venusta* [18, 56] and *C. lutrensis* [51], and characid species [57, 58]. In addition, partial morphological similarities were determined between the lentic and lotic (above the barrier site) populations of *P. antalyae*. Gene flow between two populations was probably the most influential mechanism underlying the absence of clear morphological differentiation between two populations. Thus, the shape similarities and differences in this study could be the results of phenotypic plasticity under gene flow which can significantly restrict morphological differentiation.

Body shapes of the populations from lotic sites 1 and 2 differed significantly. Although these two sites were in the stream channel, that is, the lotic system, they differ in terms of habitat characteristics (Table 1). While site 1 is in a small man-made concrete channel, site 2 is a natural stream bed. However, there are also natural and artificial barriers (HPPs and waterfalls) between the two sites. The population from lotic site 2 was represented by a larger head, a narrower caudal peduncle, and a deeper body as opposed to the population from site 1. Previous studies have reported that geographic distances or physical barriers (HPPs (constructed in 1966) and waterfall) between habitats [59, 60] pose a major obstacle to gene flow [61; 62], hence increasing intraspecific morphological differences in various fish species [16, 17, 63]. This situation reduces the genetic diversity of populations above the barrier and even increases the genetic differentiation between populations above and below the barrier [64]. Düden Stream basin is in a karst region. Karst regions consist of irregular limestone and/or dolomite rocks and contain sinkholes, sinking streams, caves, and well-developed underground drainage systems throughout the region. In such systems, there is usually a strong interaction between surface and groundwater [65]. It is known that in the karst environment, small-sized cyprinid fish (*Delminichthys adspersus*) frequently migrate and spend several months underground [66]. However, in the Düden Stream, there is an HPP before the water first disappears into the sinkhole and a waterfall beyond where it last emerged. Unfortunately, since we do not have genetic data, we cannot know whether there is gene flow between populations through the underground stream network. Therefore, it is unclear whether the main driving force behind morphological variation between populations was genetic differentiation, phenotypic plasticity due to habitat differences, or a combination of both.

Both univariate and multivariate analyses revealed significant differences between lentic and lotic site 2. Before comparing the shape of populations, it is necessary to define the habitat characteristics well. While the lentic site presents a “natural” small lake habitat, lotic site 2 was a heavily “anthropogenically” degraded stream habitat because it was located in the Antalya city center (the population is 2.688,004) and was frequently exposed to industrial and domestic waste (water quality was classified as “very poor” according to HPI [21]). Fish can respond quickly to rapid natural or anthropogenic environmental changes [7]. The reason for the morphological difference between both populations might be due to the change in habitat type (lentic vs. lotic) and related environmental parameters. As an endemic fish species, it would be beneficial for the sub-population of *P. antalyae* to have such plastic characteristics that allow them to cope with such an unstable, anthropogenically disturbed environment, where water quality parameters likely change abruptly [65].

## Conclusion

Fish can migrate long distances within stream systems to reproduce, feed, and escape habitat changes. Physical barriers are not required to prevent gene flow between populations; moreover, pre- and post-zygotic mate selection, local differences in water chemistry, or other environmental conditions can create barriers for fish coming from different habitats [64].

In this study, although there were no significant differences among sexes in both the external morphology and functional traits of *P. antalyae* which is an endemic fish, along the Düden Stream, it was revealed that there were significant differences between the populations. The reason for these differences determined in the morphology and traits of the populations may depend on habitat type (lotic vs. lentic), ecological (food preferences, foraging tactics), environmental (water quality parameters and substrate structure, etc.) and obstruction of gene flow. Therefore, future studies should consider determining the evolutionary and ecological mechanisms (genetic, and ecological) underlying the inter-population morphological variation, as well as to what extent, if any, they represent ecological specialization.

## Data Availability

All data analyzed during this study are included in this article and can be obtained from the corresponding author if needed.
